# The Underlying Effect of Urate Levels on Female Infertility

**DOI:** 10.3390/metabo14100564

**Published:** 2024-10-21

**Authors:** Muhammad Naveed, Jennifer W. Hill

**Affiliations:** 1 Department of Physiology and Pharmacology, School of Medicine and Life Sciences, University of Toledo, Toledo, OH 43614, USA; drnoveed@aol.com; 2 Center for Diabetes and Endocrine Research, University of Toledo, Toledo, OH 43614, USA

Female infertility is a complex and multifaceted condition that affects millions of women globally. Its causes vary, ranging from hormonal dysregulation to structural abnormalities due to genetic, lifestyle, and environmental factors [1]. The most-studied endocrine contributors include conditions like polycystic ovary syndrome (PCOS) [2] and ovulatory dysfunction [3] (Figure 1). Structural abnormalities affecting the fallopian tube can result from pelvic inflammatory disease (PID), endometriosis, or prior surgeries that hinder the transport of the oocyte or embryo, preventing fertilization or implantation. Cervical factors, including cervical stenosis, mucus issues, or infections, may impede sperm mobility and survival, preventing fertilization. Finally, vaginal factors, including infections or structural abnormalities, can obstruct sperm transport or create an inhospitable pH balance, complicating conception. By understanding these distinct reproductive barriers, clinicians can better diagnose and treat infertility issues related to tubal, cervical, and vaginal conditions, improving reproductive outcomes.

Recent genetic and metabolic research advances have led to a growing interest in metabolic biomarkers that may also play a pivotal role in reproductive health [4]. One such biomarker is urate, the final oxidation product of purine metabolism. Elevated urate levels have long been associated with gout, cardiovascular disease (CVD), and metabolic syndrome [5]. However, a new study utilizing Mendelian randomization (MR) has provided evidence of a causal link between elevated urate levels and female infertility [6]. This editorial explores the implications of this pioneering research, discussing how urate levels may influence female reproductive health, the significance of using MR in fertility studies, and the potential for new interventions to improve fertility outcomes for women.

Urate is an organic compound that plays a dual role in human physiology, acting as both an antioxidant and a pro-oxidant, depending on its concentration and the tissue environment. Urate is a powerful antioxidant at low to moderate levels, protecting cells from oxidative damage. However, it becomes a pro-oxidant at elevated concentrations, contributing to oxidative stress, inflammation, and the formation of uric acid crystals, leading to conditions like gout [7]. In recent years, the role of urate in cardiovascular health, metabolic syndrome, and chronic kidney disease (CKD) has also been identified [8]. However, its potential role in reproductive health is still being investigated. Studies have linked elevated urate levels to metabolic conditions such as insulin resistance [9], obesity [10], and type 2 diabetes [11], all of which are also closely associated with female reproductive disorders like PCOS and infertility [12]. Elevated urate levels in the bloodstream may influence systemic inflammation and oxidative stress, adversely affecting reproductive tissues, ovarian function, and hormonal balance [13]. Given the shared metabolic pathways between infertility and chronic metabolic diseases, understanding the influence of urate on female infertility is critical.

Establishing causality in these relationships has been challenging in observational studies [14]. This is where MR provides an advantage. MR is a genetic epidemiological method that uses genetic variants as instrumental variables to study the relationship between a risk factor (e.g., urate levels) and an outcome (e.g., female infertility). MR mitigates confounding factors and prevents reverse causality by leveraging genetic variants that are randomly inherited [15]. In other words, MR studies take advantage of genetic variants fixed at conception, mimicking the structure of a randomized controlled trial. Thus, MR enables researchers to determine whether genetically elevated urate levels directly cause infertility or if they are associated with other metabolic conditions that heighten the risk of infertility.

A study on MR by Hong and colleagues investigated whether elevated urate levels have a causal effect on female infertility in the USA [16]. This study found a significant causal link between elevated urate levels and a higher likelihood of female infertility. This finding suggests that urate is not merely a byproduct of metabolic dysfunction, but could actively contribute to the physiological changes that impair female reproductive function. By analyzing genetic variants known to influence urate levels, the researchers assessed whether women with genetically higher urate levels were at an increased risk of infertility. Several mechanisms may explain this relationship, ranging from oxidative stress and inflammation to insulin resistance and neuroendocrine dysregulation.

Oxidative stress is a critical factor in the deterioration of reproductive health, affecting ovarian reserves, follicular development, and oocyte quality [17]. As a pro-oxidant in higher concentrations, elevated urate levels can contribute to oxidative stress in reproductive tissues. Excessive oxidative damage in the ovaries can impair follicular development, leading to poor oocyte quality and reduced fertility. Research suggests that oxidative stress may disrupt key signaling pathways in the ovarian follicle, impairing its ability to mature correctly [18]. In turn, this can lead to a reduction in viable oocytes, hampering the woman’s ability to conceive naturally or through assisted reproductive technologies.

Chronic low-grade inflammation is another common factor in both metabolic diseases and infertility [19]. Urate stimulates inflammation by activating immune cells and releasing pro-inflammatory cytokines [20]. This inflammatory environment might throw off the delicate balance of reproductive hormones like estrogen, follicle-stimulating hormone (FSH), and luteinizing hormone (LH), which control ovarian function [21]. Any resulting irregular ovulation or anovulation would lead to infertility. Inflammatory factors in the reproductive system may also contribute to conditions like endometriosis and PCOS, further complicating fertility [22]. Elevated urate levels are often linked to insulin resistance, resulting in elevated blood glucose levels, a crucial feature of PCOS [23]. This common endocrine disorder affects up to 10% of women of reproductive age and is one of the leading causes of female infertility. Insulin resistance can increase androgen levels and disturb LH levels, disrupting ovulation and menstrual regularity [24]. Given the close relationship between elevated urate levels, insulin resistance, and PCOS, managing urate levels could help mitigate some of the fertility challenges posed by PCOS.

Hyperuricemia leads to oxidative stress and systemic inflammation, contributing to the development of insulin resistance. Insulin resistance, in turn, worsens metabolic-associated steatotic liver disease (MASLD), also known as non-alcoholic fatty liver disease (NAFLD), by promoting liver fat accumulation while simultaneously contributing to reproductive dysfunction through hormonal disruption. MASLD exacerbates metabolic dysregulation and may further impair reproductive health by affecting the liver’s role in hormone metabolism and systemic inflammation.

Urates may also impact fertility because of their effects on vascular health. Researchers have linked high urate levels to endothelial dysfunction [25], which impairs blood flow and may alter the uterine environment, resulting in poor endometrial receptivity [26] and implantation failure. Proper blood flow is crucial for developing a healthy endometrium, embryo implantation, and early pregnancy maintenance.

Uric acid (urate) can impact both male and female reproductive health, but has a greater effect on female infertility because of its relationship with oxidative stress, insulin resistance, and hormonal regulation, which all play significant roles in female reproductive health. Moreover, hyperuricemia can significantly impact conditions such as PCOS, insulin resistance, and MASLD, which are directly associated with reproductive dysfunction in women and have a pronounced impact on female infertility compared to males. Additionally, the endometrium and ovaries are particularly sensitive to oxidative damage, making hyperuricemia a potential contributor to reproductive challenges unique to women.

The findings from this MR study open new avenues for managing female infertility by highlighting urate as a potentially modifiable risk factor. Confirmation of elevated urate levels as a causal factor in female infertility would have profound clinical implications. Women struggling with infertility, particularly those with metabolic conditions like PCOS or insulin resistance, could consult their healthcare provider about including the use of pharmacological agents that reduce urate levels, such as allopurinol or febuxostat [27]. These medications are already widely used to treat gout, and could offer an additional benefit to reproductive health. Lifestyle interventions that reduce urate levels—such as dietary changes, increased physical activity, and weight loss—could also become part of an individual’s fertility management strategy [28]. Finally, women trying to conceive could limit foods high in purines, such as red meat, organ meats, and alcohol, as they are known to elevate urate levels [29]. Meanwhile, increasing the intake of foods rich in antioxidants, like fruits and vegetables, may help reduce oxidative stress and improve reproductive outcomes [30]. Routine screening of urate levels in women experiencing infertility could help identify those at risk and guide individualized treatment plans.

Although this work offers strong evidence for the causal involvement of urate in female infertility, more investigation is required to clarify the exact molecular mechanisms at play. Longitudinal studies and clinical trials examining how lowering urate affects fertility would be very helpful in confirming these results and developing evidence-based guidelines for managing urate levels in reproductive health. Moreover, future research should explore whether the effect of urate on infertility varies across different subgroups of women. For instance, does urate have a more pronounced impact on fertility in women with PCOS compared to those with unexplained infertility? Understanding these merits could lead to more personalized approaches to treatment.

The link between urate levels and female infertility, as revealed through MR, represents a significant step forward in our understanding of reproductive health. This study establishes a causal relationship between elevated urate levels and infertility, highlighting a new metabolic factor that could improve fertility outcomes. Whether through lifestyle changes, pharmacological interventions, or routine screening, managing urate levels may soon become an essential consideration in fertility treatment protocols. The relationship between metabolic health and reproductive health becomes increasingly apparent as we continue to unravel the complexities of female infertility. Addressing metabolic factors like urate could enhance fertility and improve the overall health and well-being of women of reproductive age.

## Figures and Tables

**Figure 1:**
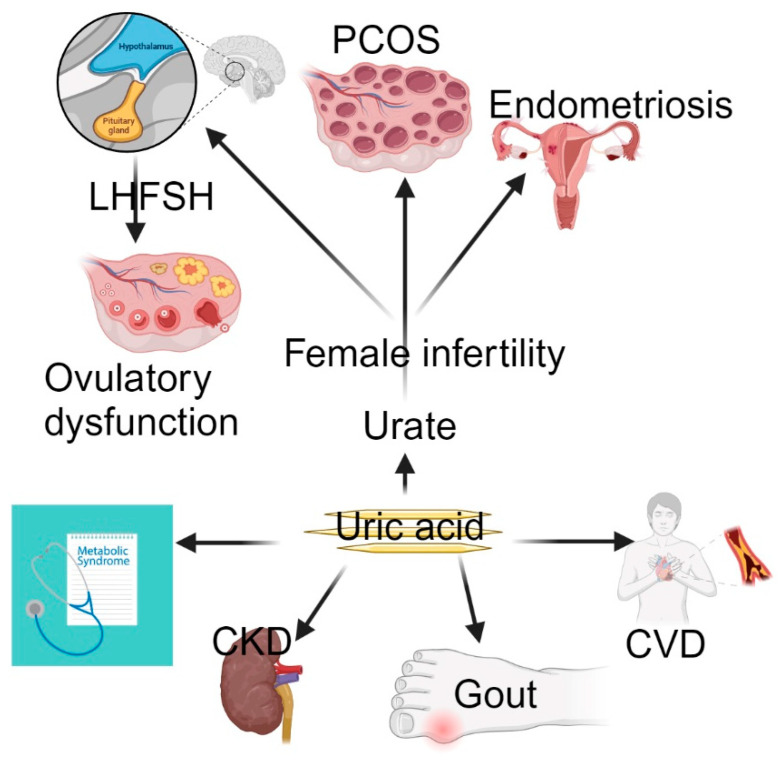
Uric acid causes gout, CVD, CKD, and metabolic syndrome. However, MR has provided evidence of a causal link between elevated urate levels and female infertility. *Abbreviations*: CKD, chronic kidney disease; CVD, cardiovascular disease; PCOS, polycystic ovary syndrome; FSH, follicle-stimulating hormone; LH, luteinizing hormone.
